# Sex differences in time trends of colorectal cancer in England and Wales: the possible effect of female hormonal factors.

**DOI:** 10.1038/bjc.1996.120

**Published:** 1996-03

**Authors:** I. dos Santos Silva, A. J. Swerdlow

**Affiliations:** Epidemiological Monitoring Unit, Department of Epidemiology and Population Sciences, London School of Hygiene and Tropical Medicine, UK.

## Abstract

Differences between the sexes in time trends of colorectal cancer incidence 1962-87 and mortality 1960-91 in England and Wales are examined in relation to changes in female hormonal factors. There was a trend in the sex ratio of this tumour, particularly marked for the descending colon, whereby the female excess in risk at young ages has almost disappeared but the male excess at older ages has increased. This trend started for cohorts born since the 1920s and coincided with the increase in the use of oral contraceptives and, to a lesser extent, with increases in fertility. The decline has been particularly pronounced for women at young ages born since 1935-39, coinciding with the spread of oral contraceptive use to younger age groups. These results are consistent with the hypothesis that female hormonal factors may play a role in the aetiology of colorectal cancer and with the possibility that oral contraceptive use might exert a protective effect in the descending colon.


British Journal of Cancer (1996) 73, 692-697
© 1996 Stockton Press All rights reserved 0007-0920/96 $12.00

### Sex differences in time trends of colorectal cancer in England and Wales: the possible effect of female hormonal factors

I dos Santos Silva and AJ Swerdlow

*Epidemiological Monitoring Unit, Department of Epidemiology and Population Sciences, London School of Hygiene and Tropical Medicine, Keppel Street, London WC1E 7HT, UK.*

**Summary** Differences between the sexes in time trends of colorectal cancer incidence 1962-87 and mortality 1960-91 in England and Wales are examined in relation to changes in female hormonal factors. There was a trend in the sex ratio of this tumour, particularly marked for the descending colon, whereby the female excess in risk at young ages has almost disappeared but the male excess at older ages has increased. This trend started for cohorts born since the 1920s and coincided with the increase in the use of oral contraceptives and, to a lesser extent, with increases in fertility. The decline has been particularly pronounced for women at young ages born since 1935-39, coinciding with the spread of oral contraceptive use to younger age groups. These results are consistent with the hypothesis that female hormonal factors may play a role in the aetiology of colorectal cancer and with the possibility that oral contraceptive use might exert a protective effect in the descending colon.

**Keywords:** colon cancer; rectal cancer; sex ratio; fertility; oral contraceptive; hormone

Prompted by the observation that there were marked age-specific differences between men and women in the risk of colorectal cancer McMichael and Potter (1980) raised the hypothesis that reproductive factors may play a role in the aetiology of this cancer in women. The epidemiological evidence from analytical studies on this hypothesis was recently reviewed by La Vecchia and Franceschi (1991). Briefly, some analytical studies (e.g. Kravdal *et al.*, 1993; Potter and McMichael, 1983) have found that parity decreases the risk of colorectal cancer, which is consistent with the hypothesis. Others (e.g. Chute *et al.*, 1991), however, have not shown such as association, and in one study (Kune *et al.*, 1989) an association with number of children was also present in males, leading to the suggestion that this association is due to a lifestyle factor, not a female reproductive/hormonal factor. Relations to other reproductive events, such as age at first birth, age at menarche, age at menopause and exogenous oestrogen use have also been inconsistent (La Vecchia and Franceschi, 1991).

Since there have been marked changes in fertility and oral contraceptive use between successive generations of women born in England and Wales, differences in time trends in the risk of these tumours between the sexes might be expected if reproductive-related factors played a role in aetiology in females. In this paper, recent sex-specific time trends in the incidence and mortality of colon (overall and by subsite) and rectal cancers in England and Wales are examined in relation to changes in reproductive variables.

### Materials and methods

Data on colorectal cancer (International Classification of Diseases, ICD7-9: 153 and 154) (WHO, 1957, 1967, 1977) incidence 1962-87 and mortality 1960-91 were extracted respectively, from the Office of Population Censuses and Surveys (OPCS) cancer registration files and national mortality files. Mid-year population estimates for England and Wales for the years 1960-91 were also extracted from the OPCS files. Cancer risks were analysed overall and separately for each subsite: ascending colon [including caecum and appendix (ICD7-8: 153.0; ICD9: 153.4, 153.5, 153.6)], transverse colon [including hepatic and splenic flexures (ICD7-8: 153.1; ICD9: 153.0, 153.1, 153,7)], and descending colon [including sigmoid colon (ICD7-9: 153.2, 153.3)]. Since the proportion of colon cancer deaths of subsite unknown increased suddenly in 1984 when OPCS stopped requesting further information from certifiers if the subsite of origin was omitted from the death certificate (Swerdlow, 1989), analyses of mortality by colon subsite had to be restricted to the years 1960-83. Secular trends were assessed by fitting a Poisson regression model (Breslow and Day, 1987); results are reported as average annual percentage changes in the incidence and mortality rates during the period. Sex ratios were calculated as female-male (FM) incidence (or mortality) rate ratios. Data on female reproductive variables for successive cohorts of women born in England and Wales were extracted and recalculated as in dos Santos Silva and Swerdlow (1995).

To compare changes in cancer risk for successive birth cohorts with changes in reproductive behaviour, age-standardised cohort registration ratios (SCRRs) for each sex were calculated by the indirect method (Beral, 1974), using the average age-specific rates for the entire period in each sex to derive the expected values for each cohort. These cohort ratios are a summary measure of the risk experience of each generation for the ages included in the study, relative to the same ages for all cohorts included in the analysis, after adjusting for differences in the age structure. Although it would be possible to calculate a summary measure of the sex ratio for each cohort, this measure would have been misleading since the age groups included in the analysis differed from cohort to cohort and the size and direction of the FM rate ratios varied substantially with age. As a result, this all-age summary measure would change between successive cohorts even in the absence of any real changes, because it would comprise different age groups in the different cohorts. Instead, we compared cohort changes in reproductive behaviour directly with changes in the female SCRRs calculated separately for two different age groups (0-44 and 45-84 years). Since we are comparing cohort (not cross-sectional) changes in reproductive behaviour with cohort changes in cancer risk, there is no need to build up any time lag. Ninety-five per cent confidence intervals for the SCRRs were estimated by using approximate methods based on the normal distribution or, when the number of cancers underlying these ratios was less than 20, exact methods based on the binomial distribution.

Correspondence: I dos Santos Silva
Received 24 March 1995; revised 14 September 1995; accepted 4 October 1995

### Results

In each sex, trends in colon cancer incidence (without dividing by subsite) differed by age (Table I). At ages under 45 years the incidence of this tumour declined during the study period, whereas at older ages there were increases in risk. Although the directions of these changes were similar for males and females, there were sex differences in their magnitude. The decline in incidence at young ages was more marked in females, whereas the increase at older ages was more pronounced in males. As a result, there was a change in the sex ratio by age whereby the female excess in risk at young ages present in the early years of the study period almost disappeared, and the male excess at older ages increased further (Figure 1a).

Analyses by colon subsite showed patterns in the same direction as those for the colon overall, but differing in degree. For each subsite there were declines in risk at ages 0–44 (except for the descending colon in men) and increases at older ages, but to a different extent for males and females (Table I). The greatest change in the shape of the sex ratio curve occurred for the descending colon. In this segment, there was a marked decline in the female excess in risk at ages under 45 years (Figure 1d), due to pronounced decreases in risk in women but not men. In the ascending colon, there were no clear changes in the sex ratio at young ages, but the slight female excess at ages 45–74 present in 1962–66 was gradually replaced by a slight male excess (Figure 1b). In the transverse colon, there was a slight decrease in the sex ratio at ages 50 and over and no appreciable change at younger ages (Figure 1c).

The incidence trends for cancer of the rectum were in the same direction as those observed for colon cancer, but both the declines in risk at young ages and the rises at older ages were less marked (Table I). The trends in the sex ratio were also less pronounced than for colon cancer; there was a decrease at ages 35–54 years but no clear changes at other ages (Figure 1e).

Analyses by year of birth (Figure 2) showed that the progressive decline in the sex ratio for colon cancer (all subsites) was particularly marked for generations born since 1920–24 (for simplicity, only risks for alternate cohorts were plotted in Figure 2). This trend was most pronounced for the descending colon (Figure 2d). For the ascending colon, the declines in the sex ratio were much less marked than for the descending colon and started with earlier cohorts (those born at the turn of the century) (Figure 2b). For the transverse colon, the pattern was irregular, with no consistent changes over time (Figure 2c). There was a downward trend in the sex ratio of rectal cancer incidence for cohorts born from 1915–1939, but no consistent trend for persons born thereafter (Figure 2e).

Mortality data showed sex differences in colon cancer trends similar to those observed for incidence (not shown in figures). The major changes in the sex ratio occurred in the descending colon, where the female excess at ages under 60 years present in the earlier years of the study period almost disappeared and the male excess at older ages increased slightly. This decline in the sex ratio was particularly pronounced for cohorts born since 1920–24. There were also decreases in the sex ratio of rectal cancer for cohorts born from 1905 to 1939, but they were much less marked than for colon cancer.

Figure 3 shows female cohort trends in the risk of developing cancer in the descending colon, the subsite for which the changes in the sex ratio were most pronounced, in relation to changes in reproductive behaviour. The increase in the cancer risk for successive generations of women born before 1920 (Figure 3a and b) were accompanied by marked declines in family size (Figure 3c), although the level of childlessness remained practically constant (Figure 3d). The decline in cancer risk for cohorts of women born from 1920–24 to the mid-1940s coincided with a pronounced decrease in nulliparity, a slight decline in mean age at first birth and an increase in family size. However, cancer incidence continued to decline for women born after 1940 despite falls in their fertility. The marked decline in the cancer risk for cohorts born since 1920–24 coincided with the increase in use of oral contraceptives (Figure 3a and b). At ages 0–44 years, the fall in the cancer risk was more marked for women born since 1935–39, who were the ones who started to use oral contraceptives at young ages (Figure 3b).

**Table I** Mean annual percentage change in incidence rates of colorectal cancer in England and Wales, 1962–87

| *Site* | *Age (years)* | *Mean annual change (%)* *Males* | *Females* |
|---|---|---|---|
| Colon (all subsites) | All ages | +1.35 | +0.80 |
| | 0–44 | −0.66 | −1.75 |
| | 45+ | +1.54 | +1.02 |
| Ascending colon | All ages | +2.27 | +1.52 |
| | 0–44 | −1.01 | −2.27 |
| | 45+ | +2.52 | +1.74 |
| Transverse colon | All ages | +0.65 | +0.22 |
| | 0–44 | −1.02 | −0.84^NS^ |
| | 45+ | +0.86 | +0.43 |
| Descending colon | All ages | +1.29 | +0.62 |
| | 0–44 | +0.15^NS^ | −1.28 |
| | 45+ | +1.46 | +0.91 |
| Rectum | All ages | +0.50 | +0.48 |
| | 0–44 | −0.37^NS^ | −0.47 |
| | 45+ | +0.65 | +0.69 |

All values were statistically significant at the 0.05 level except those indicated by ^NS^.

### Discussion

Potential artefacts need to be considered when interpreting the data. Firstly, it is unlikely that the use of diagnostic tests (e.g. endoscopy and barium enema) was sex related, and any secular changes in the diagnosis, treatment and registration of these cancers should have affected the two sexes similarly. This is confirmed by the fact that the trends in the sex ratios were similar for incidence and mortality data. Second, the proportion of colon cancers of undefined location was of similar magnitude in the two sexes (about 26% in each sex for the incidence data and 22% for the mortality data) and did not change appreciably over time. Therefore, it is unlikely that it would have affected the sex differences in the time trends shown here. Moreover, similar sex differences were present when colon cancer of all subsites was considered (Figures 1 and 2).

McMichael and Potter (1980) observed a similar change in the sex ratio of colon (all subsites) cancer incidence and mortality between the early 1960s and the early 1970s in various developed countries (including England and Wales) but did not analyse by subsite. Similar sex differences in colorectal cancer mortality trends have also been shown in several countries from 1959 to 1986 (Hoel *et al.*, 1992) but again without analyses by subsite. Data from the Connecticut Tumour Registry (USA) for the years 1950–84 showed declines in the incidence of cancer of the descending colon for women born since the 1910s but not for men (Dubrow *et al.*, 1993). To our knowledge, this is the only previous study to have examined trends in the sex ratio of this tumour by subsite.

The trend in the sex ratio observed in the present study suggests that female reproductive factors might have been important in aetiology. Another possibility is that the changes might have been due to sex differences in exposure to dietary factors or other sex-shared factors. Alcohol intake is the potentially aetiological dietary exposure for which male–female differences are likely to be most marked. No sex-specific data on alcohol intake are available, but trends in mortality from cirrhosis of the liver and alcoholism, and trends in drunkenness offences, which closely parallel the trend in alcohol consumption (Donnan and Haskey, 1977), have been similar for men and women despite sex differences in the absolute levels (Donnan and Haskey, 1977). The proportions of moderate and heavy drinkers in each sex also remained constant between 1978 and 1984 (OPCS, 1986). These data do not indicate recent differences between the

**Figure 1** Female to male (FM) incidence rate ratios for colorectal cancer, England and Wales, 1962–87, by year of incidence, age and subsite. (**a**) Colon (all subsites). (**b**) Ascending colon. (**c**) Transverse colon. (**d**) Descending colon. (**e**) Rectum.

sexes in trends in alcohol consumption. Data on sex-specific trends in the intake of other foods and nutrients are scarce. The National Food Survey does not distinguish between the food consumption of men and women (Ministry of Agriculture, Fisheries and Food; MAFF, 1991). In a recent compilation of all British studies in which measurements of individual fat intake were carried out there was, however, no evidence that fat intake has been decreasing more in women

**Figure 2** Female to male (FM) incidence rate ratios for colorectal cancer, England and Wales, 1962–87, by year of birth, age and subsite. (**a**) Colon (all subsites). (**b**) Ascending colon. (**c**) Transverse colon. (**d**) Descending colon. (**e**) Rectum.

**Figure 3** Incidence trends of cancer of the descending colon in women ages (**a**) 45–84 years and (**b**) 0–44 years in relation to cumulative percentage of ever users of oral contraceptives under ages 25 and 35, (**c**) average family size, mean age at first birth and (**d**) childlessness for successive cohorts born 1875–1959, England and Wales (Data on mean age at first birth are only available for cohorts born since 1920).

than in men (Stephen and Sieber, 1994). There are no data available on sex-specific trends in dietary fibre intake but recent cross-sectional studies did not show any consistent differences in fibre intake between men and women (Bingham and Cummings, 1980). A sedentary lifestyle has been associated with an increased risk of colon (but not rectal) cancer in some studies (e.g. Lee *et al.*, 1991), but data on sex-specific trends of physical exercise are not available.

Cholecystectomy, which alters the bile acid composition of the intestine, has been linked to an increased risk of right-sided colon cancer (Lino *et al.*, 1981; Vernick and Kuller, 1981). Although the frequency of this type of surgery has increased over time in England and Wales, the sex differences have decreased. This operation was three times more common in women than in men in 1961 (Ministry of Health and General Register Office, 1964) but by 1985 this ratio had declined to two (Department of Health and Social Security, and Office of Population Censuses and Surveys, 1987). This decline in the sex ratio of cholecystectomies could partly explain the observed fall in the female excess in the risk of cancer in the ascending colon during the study period.

The decline in the sex ratio for cohorts born from 1915 to 1919 to the mid-1940s paralleled increases in female fertility. For cohorts born since the 1940–44 cohort, fertility has been declining whereas the colon cancer sex ratio has continued to fall. There is some evidence that the downward trend in the sex ratio for rectal cancer, however, might have been reversed for generations born since 1945–49. Oral contraceptive use increased progressively for successive cohorts of women born since the 1920s, but so far there is little evidence from analytical studies that oral contraceptives protect against colorectal cancer. Of the four case–control studies that have examined this relationship only one (Potter and McMichael, 1983) indicated a protective effect (although not statistically significant) of oral contraceptives on the risk of colon and to a lesser extent rectal cancers, but the number of users was small and no analyses by colon subsite were carried out. In the only case–control study that conducted analyses by colon subsite (Peters *et al.*, 1990), no protective effect was found for any of the subsites. Results from the Nurses Health Study Cohort (Chute *et al.*, 1991), the only prospective study to have addressed this issue, showed a slight inverse association with colon cancer overall and a positive association with rectal cancer but no obvious trend with duration of use. Further analyses by colon subsite did not reveal any clear pattern but the numbers of cases were small. In summary, very few analytic studies have examined the relationship between oral contraceptive use and risk of colorectal cancer and those that did were based on small numbers of cases by subsite and most of them did not take into account potential confounders such as diet, alcohol intake and physical exercise.

Some studies (e.g. Chute *et al.*, 1991; Jacobs *et al.*, 1994), but not all (e.g. Peters *et al.*, 1990) have shown a protective effect of hormone replacement therapy (HRT). To our knowledge there are no data available on trends in the use of HRT in England and Wales. However, most of the decline in the sex ratio observed in the present study occurred in women who were too young to have used HRT appreciably.

The decline in the sex ratio observed in the present study was most marked for the distal colon, suggesting that the role of female hormonal factors, if any, is likely to be exerted predominantly at this subsite. Results from analytic studies have been conflicting. Of the few studies that reported an association between colorectal cancer risk and female reproductive factors, some (e.g. Potter and McMichael, 1983) found that the protective effect of parity was stronger in the right than the left colon whereas others (e.g. Peters *et al.*, 1990) did not show any clear pattern among the subsites. Potter and McMichael (1983) found that the protective effect of any early age at first birth was more marked in the distal colon but another study (Howe *et al.*, 1985) showed the opposite.

The mechanisms by which female reproductive events might have a protective effect on colon cancer risks are unclear, but it has been suggested that female sex hormones might affect carcinogenesis in the large bowel by interfering with hepatic bile acid metabolism (McMichael and Potter, 1983; Potter and McMichael, 1983). The existence of oestrogen receptors in colonic tumour cells has been reported (Sica *et al.*, 1984), but their role in colon tumorigenesis is far from clear. In the present study, the potential protective role of female hormonal factors seems to be more marked in the descending segment of the colon. Since bowel subsites have different embryologic origins, blood supplies, and physiological functions (Peters *et al.*, 1990), it is biologically plausible that they might have distinct risk factors for cancer.

In summary, the sex differences in the time trends of colorectal cancer presented here are consistent with the hypothesis that female reproductive factors may play a role in the aetiology of this tumour, particularly in the descending colon. Since there have been marked changes in reproductive behaviour and oral contraceptive use in recent years it would seem worthwhile to try to clarify the role of these factors in the aetiology of this tumour, one of the most common cancers in developed countries.

### References

BERAL V. (1974). Cancer of the cervix. A sexually transmitted infection? *Lancet*, **1**, 1037–1040.

BINGHAM SA AND CUMMINGS JH. (1980). Sources and intakes of dietary fiber in man. *In Medical Aspects of Dietary Fibre*, Spiller GA and Kay RM (eds) pp. 261–284. Plenum: New York.

BRESLOW NE AND DAY NE. (1987). *Statistical Methods in Cancer Research. Vol II. The Design and Analysis of Cohort Studies*, p.136. International Agency for Research on Cancer: Lyon.

CHUTE CG, WILLET WC, COLDITZ GA, STAMPFER MJ, ROSNER B AND SPEIZER FE. (1991). A prospective study of reproductive history and exogenous estrogens on the risk of colorectal cancer in women. *Epidemiology*, **2**, 201–207.

DEPARTMENT OF HEALTH AND SOCIAL SECURITY AND OFFICE OF POPULATION CENSUSES AND SURVEYS. (1987). *Hospital In-patient Enquiry for the Year 1985. Main Tables*, Series MB4 no. 27. London: HMSO.

DONNAN S AND HASKEY J. (1977). Alcoholism and cirrhosis of the liver. *Population Trends*, **7**, 18–24.

DOS SANTOS SILVA I AND SWERDLOW AJ. (1995). Recent trends in incidence and mortality from breast, ovarian and endometrial cancers in England and Wales and their relation to changing fertility and oral contraceptive use. *Br. J. Cancer*, **72**, 485–492.

DUBROW R, BERNSTEIN J AND HOLFORD TR. (1993). Age-period-cohort modelling of large-bowel-cancer incidence by anatomic sub-site and sex in Connecticut. *Int. J. Cancer*, **53**, 907–912.

HOEL DG, DAVIS DL, MILLER AB, SONDIK EJ AND SWERDLOW AJ. (1992). Trends in cancer mortality in 15 industrilized countries, 1969–1986. *J. Natl. Cancer Inst.*, **84**, 313–320.

HOWE GR, CRAIB KJP AND MILLER AB. (1985). Age at first pregnancy and risk of colorectal cancer: a case–control study. *J. Natl. Cancer Inst.*, **74**, 1155–1159.

JACOBS EJ, WHITE E AND WEISS NS. (1994). Exogenous hormones, reproductive history, and colon cancer (Seattle, Washington, USA). *Cancer Causes and Control*, **5**, 359–366.

KRAVDAL Ø, GLATTRE E, KVÄLE G AND TRETLI S. (1993). A sub-site-specific analysis of the relationship between colorectal cancer and parity in complete male and female Norwegian birth cohorts. *Int. J. Cancer*, **53**, 56–61.

KUNE GA, KUNE S AND WATSON LF. (1989). Children, age at first birth, and colorectal cancer risk. Data from the Melbourne colorectal cancer study. *Am. J. Epidemiol.*, **129**, 533–542.

La VECCHIA C AND FRANCESCHI S. (1991). Reproductive factors and colorectal cancer. *Cancer Causes Control*, **2**, 193–200.

LEE I-M, PAFFENBARGER RS AND HSIEH C-C. (1991). Physical activity and risk of developing colorectal cancer among college alumni. *J. Natl. Cancer Inst.*, **83**, 1324–1329.

LINOS DA, BEARD CM, O'FALLON WM, DOCKERTY MB, BEART RW AND KURLAND LT. (1981). Cholecystectomy and carcinoma of the colon. *Lancet*, **2**, 379–381.

MCMICHAEL AJ AND POTTER JD. (1980). Reproduction, endogenous and exogenous sex hormones, and colon cancer: a review and hypothesis. *J. Natl. Cancer Inst.*, **65**, 1201–1207.

MCMICHAEL AJ AND POTTER JD. (1983). Do intrinsic sex differences in lower alimentary tract physiology influence the sex-specific risks of bowel cancer and other biliary and intestinal diseases? *Am. J. Epidemiol*, **118**, 620–627.

MINISTRY OF AGRICULTURE, FISHERIES AND FOOD. (1991). *Household Food Consumption and Expenditure 1990 with a Study of Trends over the Period 1940–1990*, Annual Report of the National Food Survey Committee. HMSO: London.

MINISTRY OF HEALTH AND GENERAL REGISTER OFFICE. (1964). *Report on Hospital In-patient Enquiry for the year 1961. Part II. Detailed Tables*, HMSO: London.

OFFICE OF POPULATION CENSUSES AND SURVEYS. (1986). *General Household Survey, 1984*, HMSO: London.

PETERS RK, PIKE MC, CHANG WWL AND MACK TM. (1990). Reproductive factors and colon cancers. *Br. J. Cancer*, **61**, 741–748.

POTTER JD AND MCMICHAEL JA. (1983). Large bowel cancer in women in relation to reproductive and hormonal factors: a case-control study. *J. Natl. Cancer Inst.*, **71**, 703–709.

SICA V, NOLA E, CONTIERI E, BOVA R, MASUCCI MT, MEDICI N, PETRILLO A, WEISZ A, MOLINARI AM AND PUCA GA. (1984). Estradiol and progesterone receptors in malignant gastrointestinal tumours. *Cancer Res.*, **44**, 4670–4674.

STEPHEN AM AND SIEBER GM. (1994). Trends in individual fat consumption in the UK 1900–1985. *Br. J. Nutr.*, **71**, 775–788.

SWERDLOW AJ. (1989). Interpretation of England and Wales cancer mortality data: the effect of enquiries to certifiers for further information. *Br. J. Cancer*, **59**, 787–791.

VERNICK LJ AND KULLER LH. (1981). Cholecystectomy and right-sided colon cancer: an epidemiological study. *Lancet*, **2**, 381–383.

WORLD HEALTH ORGANIZATION. (1957). *Manual of the International Statistical Classification of Diseases, Injuries and Causes of Death*, 7th revision. WHO: Geneva.

WORLD HEALTH ORGANIZATION. (1967). *Manual of the International Statistical Classification of Diseases, Injuries, and Causes of Death, 8th revision.* WHO: Geneva.

WORLD HEALTH ORGANIZATION. (1977). *Manual of the International Statistical Classification of Diseases, Injuries, and Causes of Death, 9th revision.* WHO: Geneva.


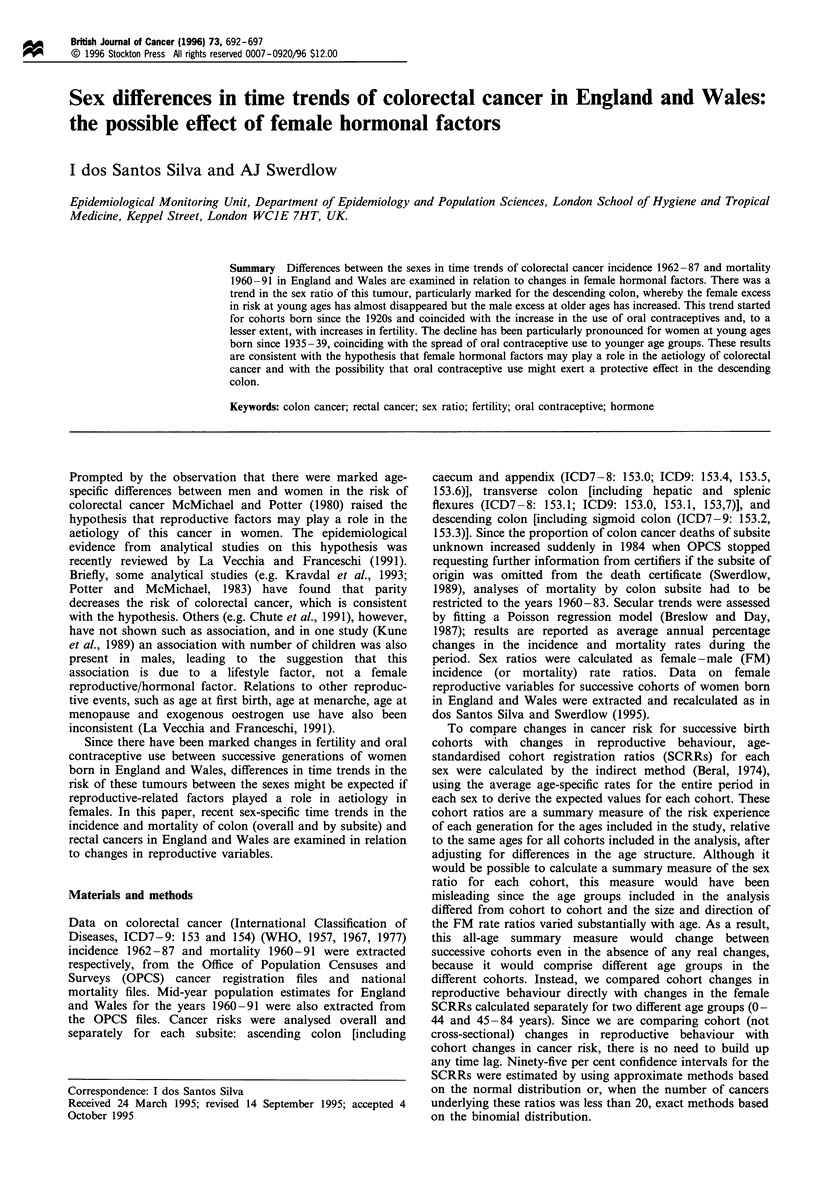

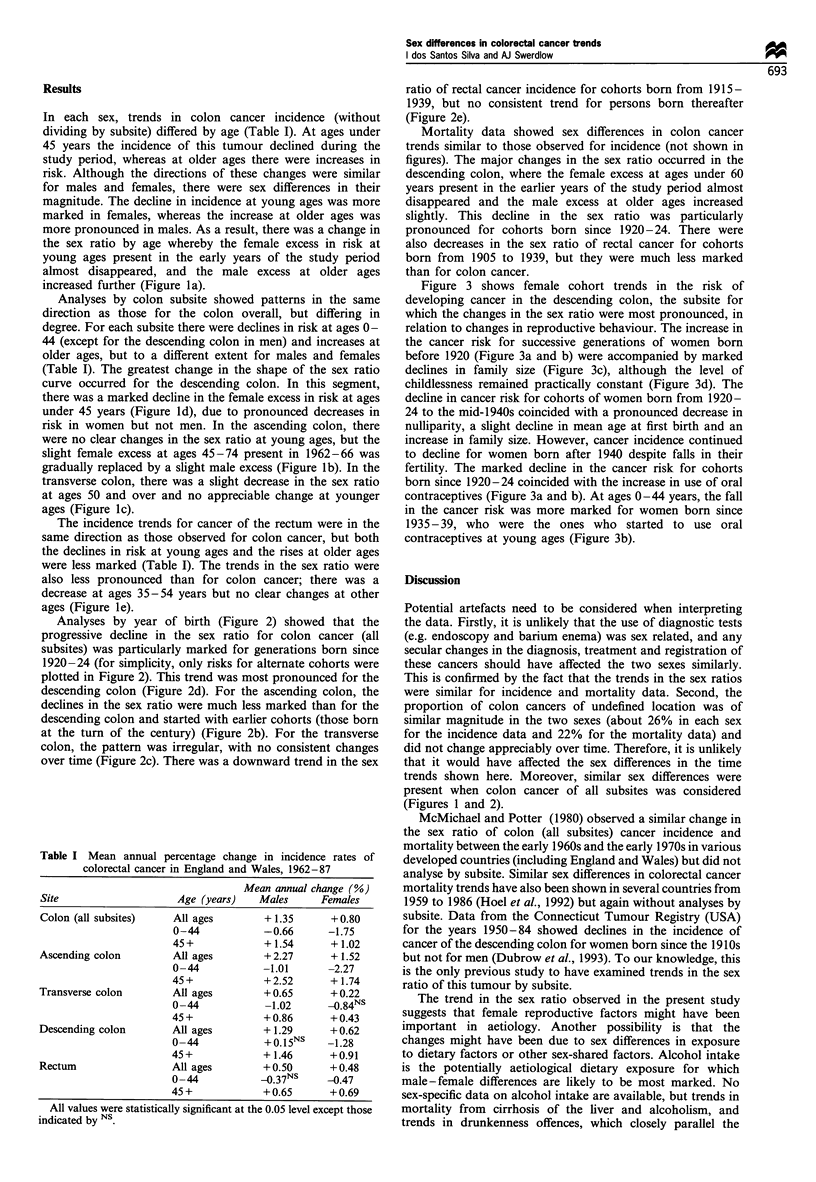

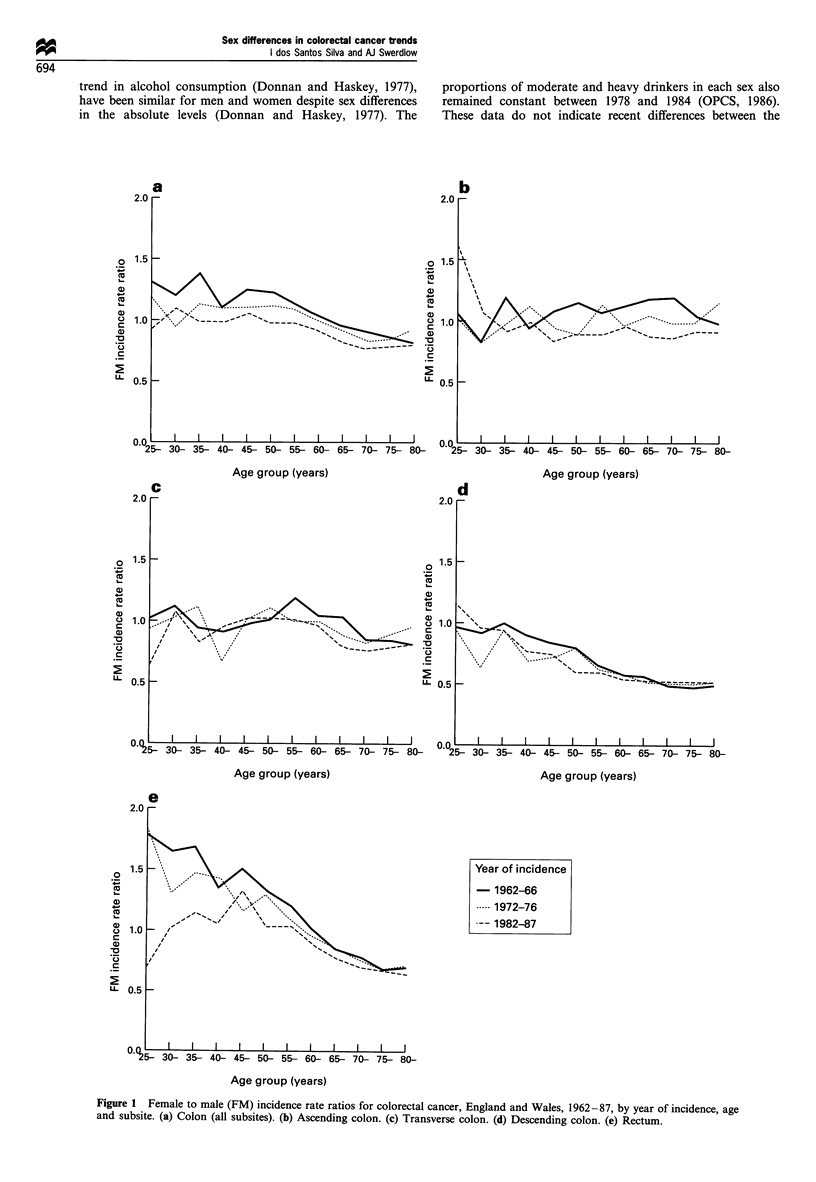

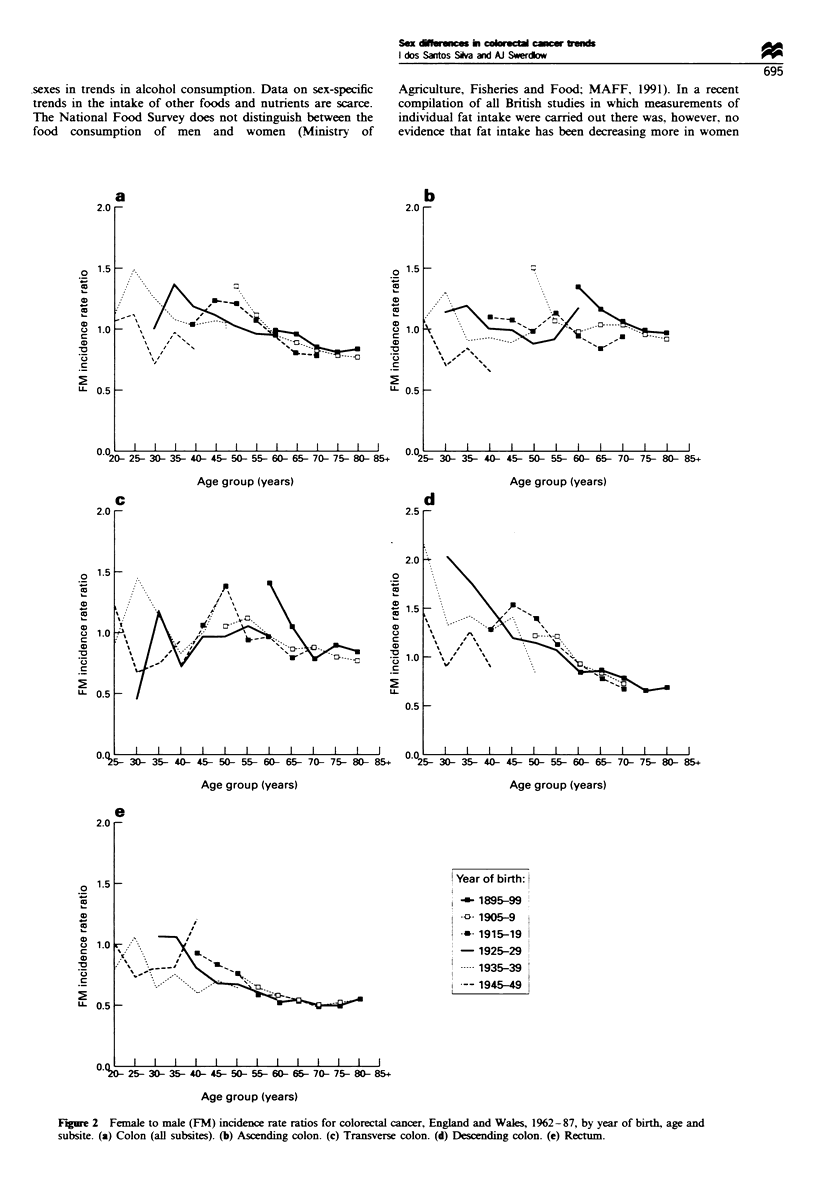

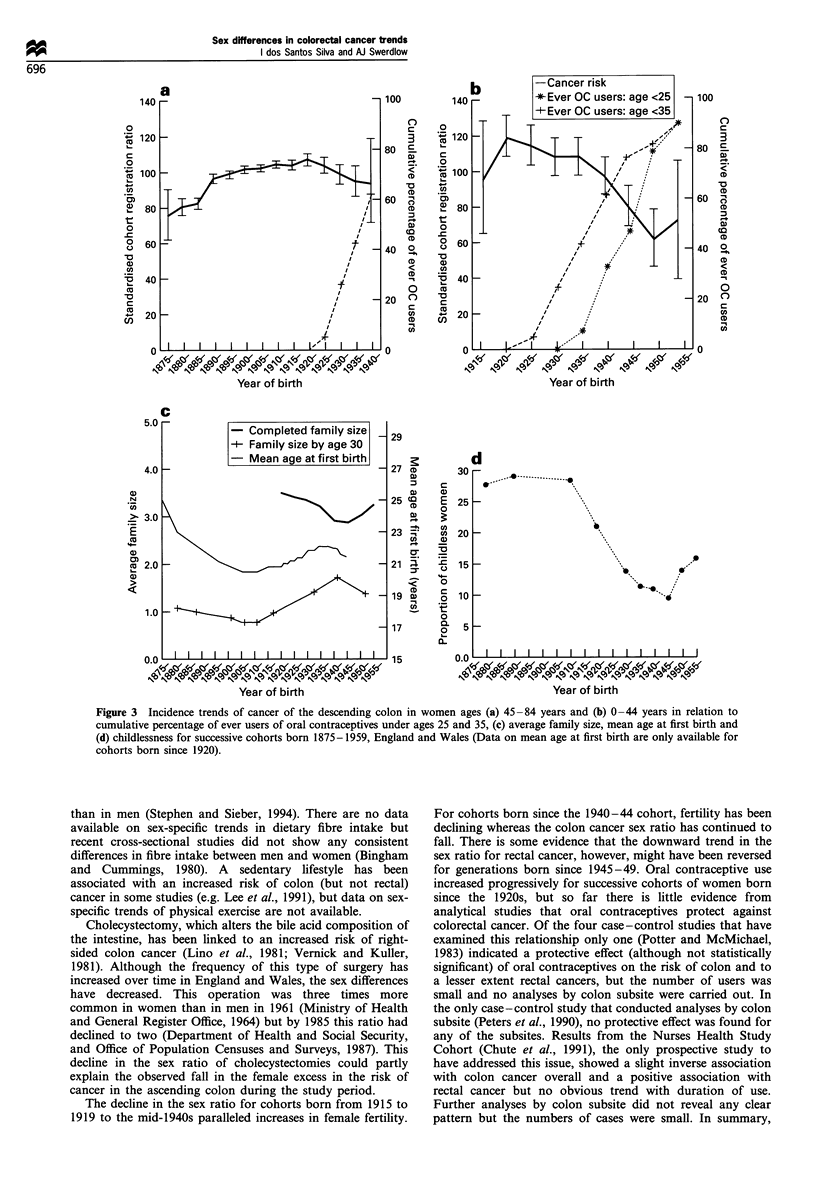

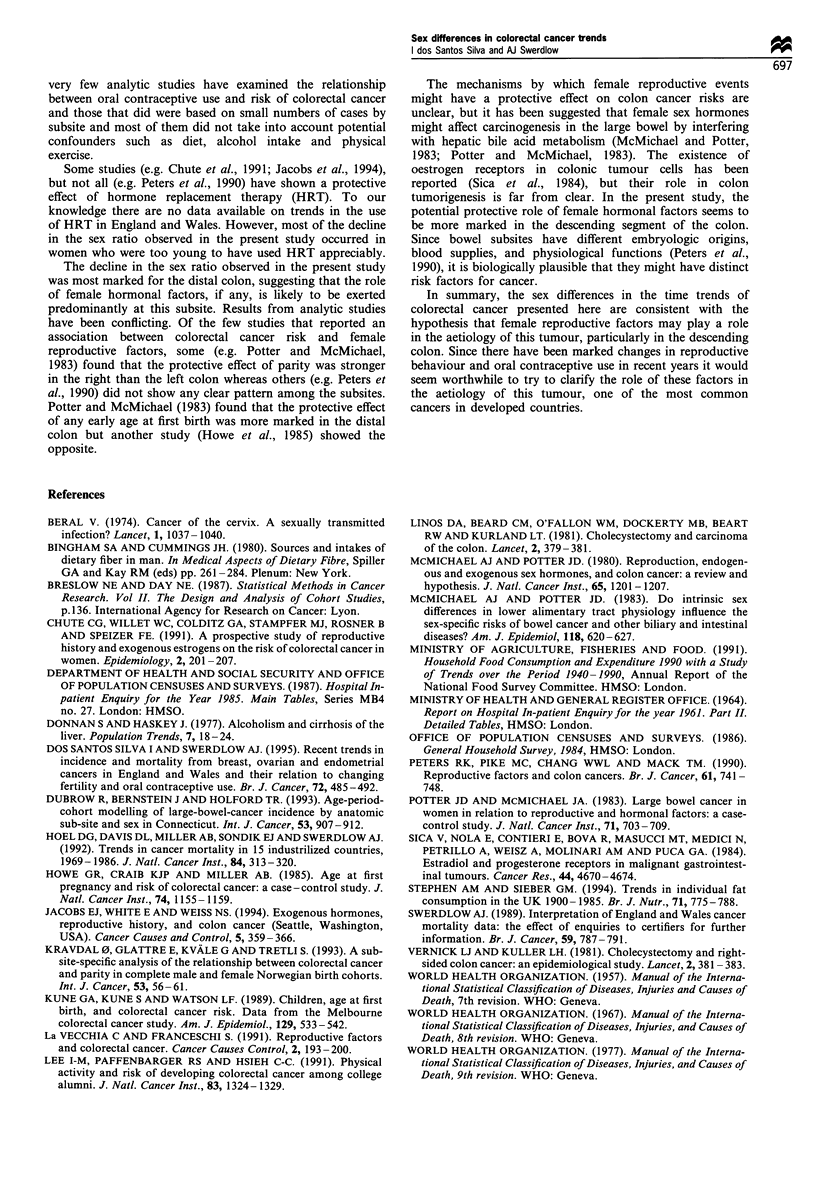


## References

[OCR_01158] Beral V. (1974). Cancer of the cervix: a sexually transmitted infection?. Lancet.

[OCR_01172] Chute C. G., Willett W. C., Colditz G. A., Stampfer M. J., Rosner B., Speizer F. E. (1991). A prospective study of reproductive history and exogenous estrogens on the risk of colorectal cancer in women.. Epidemiology.

[OCR_01196] Dubrow R., Bernstein J., Holford T. R. (1993). Age-period-cohort modelling of large-bowel-cancer incidence by anatomic sub-site and sex in Connecticut.. Int J Cancer.

[OCR_01201] Hoel D. G., Davis D. L., Miller A. B., Sondik E. J., Swerdlow A. J. (1992). Trends in cancer mortality in 15 industrialized countries, 1969-1986.. J Natl Cancer Inst.

[OCR_01204] Howe G. R., Craib K. J., Miller A. B. (1985). Age at first pregnancy and risk of colorectal cancer: a case-control study.. J Natl Cancer Inst.

[OCR_01209] Jacobs E. J., White E., Weiss N. S. (1994). Exogenous hormones, reproductive history, and colon cancer (Seattle, Washington, USA).. Cancer Causes Control.

[OCR_01216] Kravdal O., Glattre E., Kvåle G., Tretli S. (1993). A sub-site-specific analysis of the relationship between colorectal cancer and parity in complete male and female Norwegian birth cohorts.. Int J Cancer.

[OCR_01220] Kune G. A., Kune S., Watson L. F. (1989). Children, age at first birth, and colorectal cancer risk. Data from the Melbourne Colorectal Cancer Study.. Am J Epidemiol.

[OCR_01225] La Vecchia C., Franceschi S. (1991). Reproductive factors and colorectal cancer.. Cancer Causes Control.

[OCR_01231] Lee I. M., Paffenbarger R. S., Hsieh C. (1991). Physical activity and risk of developing colorectal cancer among college alumni.. J Natl Cancer Inst.

[OCR_01234] Linos D., Beard C. M., O'Fallon W. M., Dockerty M. B., Beart R. W., Kurland L. T. (1981). Cholecystectomy and carcinoma of the colon.. Lancet.

[OCR_01246] McMichael A. J., Potter J. D. (1983). Do intrinsic sex differences in lower alimentary tract physiology influence the sex-specific risks of bowel cancer and other biliary and intestinal diseases?. Am J Epidemiol.

[OCR_01241] McMichael A. J., Potter J. D. (1980). Reproduction, endogenous and exogenous sex hormones, and colon cancer: a review and hypothesis.. J Natl Cancer Inst.

[OCR_01265] Peters R. K., Pike M. C., Chang W. W., Mack T. M. (1990). Reproductive factors and colon cancers.. Br J Cancer.

[OCR_01272] Potter J. D., McMichael A. J. (1983). Large bowel cancer in women in relation to reproductive and hormonal factors: a case-control study.. J Natl Cancer Inst.

[OCR_01278] Sica V., Nola E., Contieri E., Bova R., Masucci M. T., Medici N., Petrillo A., Weisz A., Molinari A. M., Puca G. A. (1984). Estradiol and progesterone receptors in malignant gastrointestinal tumors.. Cancer Res.

[OCR_01283] Stephen A. M., Sieber G. M. (1994). Trends in individual fat consumption in the UK 1900-1985.. Br J Nutr.

[OCR_01287] Swerdlow A. J. (1989). Interpretation of England and Wales cancer mortality data: the effect of enquiries to certifiers for further information.. Br J Cancer.

[OCR_01292] Vernick L. J., Kuller L. H. (1981). Cholecystectomy and right-sided colon cancer: an epidemiological study.. Lancet.

[OCR_01188] dos Santos Silva I., Swerdlow A. J. (1995). Recent trends in incidence of and mortality from breast, ovarian and endometrial cancers in England and Wales and their relation to changing fertility and oral contraceptive use.. Br J Cancer.

